# Glioblastoma, hypoxia and autophagy: a survival-prone ‘ménage-à-trois'

**DOI:** 10.1038/cddis.2016.318

**Published:** 2016-10-27

**Authors:** Soha Jawhari, Marie-Hélène Ratinaud, Mireille Verdier

**Affiliations:** 1 EA 3842 Cellular Homeostasis and Pathologies, Faculty of Medicine, Limoges University, Limoges cedex, France

## Abstract

Glioblastoma multiforme is the most common and the most aggressive primary brain tumor. It is characterized by a high degree of hypoxia and also by a remarkable resistance to therapy because of its adaptation capabilities that include autophagy. This degradation process allows the recycling of cellular components, leading to the formation of metabolic precursors and production of adenosine triphosphate. Hypoxia can induce autophagy through the activation of several autophagy-related proteins such as BNIP3, AMPK, REDD1, PML, and the unfolded protein response-related transcription factors ATF4 and CHOP. This review summarizes the most recent data about induction of autophagy under hypoxic condition and the role of autophagy in glioblastoma.

## Facts

- Glioblastoma multiforme (GBM) is the most aggressive brain tumor. Despite its high degree of hypoxia, it can survive and resist anticancer treatments. Thus, it is important to study the main GBM adaptive strategies.
- Autophagy is a catabolic process that can be induced by hypoxia. In most cancer cases, it leads to cell survival and resistance to anticancer treatments.
- The only study about hypoxia-induced autophagy in GBM highlighted its role in cell protection against stress and in the resistance to the antiangiogenic drug bevacizumab.
- Owing to its autophagy inhibition properties, chloroquine is currently used for its antitumoral effects in GBM. Indeed, phase III clinical trials have shown an increase in median survival for patients with GBM following surgery, chemotherapy and radiotherapy.

## Open Questions

- Which signaling molecules induced by hypoxia are able to trigger autophagy?
- Why autophagy has a dual role in cancer: tumor suppression and tumor facilitation?
- Given the apparently contradictory effects of autophagy in the response of GBM to treatment (i.e., tumor cell invasiveness and senescence), how autophagy inhibition could be efficient in cancer therapy?

Gliomas originate from an uncontrolled proliferation of glial cells, and consist mainly of primary central nervous system tumors derived from astrocytes or oligodendrocytes. Several authors have successively tried to build a classification of gliomas and the scientific community currently recognizes the World Health Organization (WHO) classification^1^ that has recently been updated.^2^ This classification defines the tumor histological type according to the predominant cytological type; the tumor grade, from I to IV depends on the following criteria: increase in cell density, nuclear atypia, mitosis number, vascular hyperplasia and necrosis.^3^ Glioblastoma multiforme (GBM), a grade IV glioma,^1^ is the most common and most aggressive malignant primary brain tumor^4^ whose cell type of origin has not yet established. GBM accounts for 60–70% of all glial tumors^5^ with an incidence of 3–4 cases per 100 000 individuals per year.^6^ Palliative treatment ensures 6–9 months as median survival, which is extended to 12 months after radiotherapy and 16 months after radio-chemotherapy. Postoperative survival varies from 12 months (50%) to 24 months (20%) and reaches 36 months in 2% of cases. GBM occurs at all ages,^7^ but is more frequent between 45 and 70 years (70% of cases).^8^ It constitutes the second leading cause of cancer death in children after leukemia and the third one in adults. Death is usually due to cerebral edema, which causes an increase in intracranial pressure, and a reduced level of vigilance.^9^ GBM is a highly hypoxic tumor; deep and remote areas of the tumor suffer from a low dioxygen (O_2_) partial pressure, which can drop down to 1%. Although one would expect that this condition should slow tumor growth, cancer cells eventually develop processes allowing them not only to survive hypoxia, but also to become more aggressive. Among these adaptive responses, autophagy, a catabolic process, leads in most cases to the survival of tumor cells. This survival pathway allows the degradation of different cell components with the production of energy (adenosine triphosphate (ATP)) and metabolic precursors further recycled by the cellular anabolism.

## Glioblastoma: Molecular Alterations and Histology

GBM can appear ‘*de novo*' and quickly evolve without any prior injury; it is then called primary GBM. It can also develop slowly (5–10 years) from a low-grade astrocytoma (grade II or III), and then is called secondary GBM.^10^ Primary GBM represent over 90% of these tumors. They develop most often in patients aged over 60 years and are characterized by a brief clinical history (about 1 year after diagnosis).^10^ Secondary GBM are rare (10% of them) and occur in younger patients (average age 45). They are characterized by a longer clinical history and have a better prognosis than primary GBM.^10^ These two types of GBM are morphologically similar but present different genetic alterations (Table 1). Indeed, primary GBM are essentially characterized by an amplification of *EGFR* (epidermal growth factor receptor) (36%), a deletion of *p16InK4a* (p16 cyclin-dependent kinase inhibitor 4a) (31%) and a mutated *PTEN* (phosphatase and tensin homolog) (25%). In contrast, *TP53* (gene encoding p53) mutations are rare (30%).^11^ On the opposite, secondary GBM are primarily characterized by *TP53* mutations (65%), amplification of *EGFR* is not so frequent (8%) as well as *p16INK4a* deletion (19%) and *PTEN* mutation (4%). Monosomy 10 is observed in almost 70% of GBM, whether primary or secondary.^10^ This monosomy can affect the whole chromosome 10 or only the long arm (loss of heterozygosity 10q) especially in primary GBM.^12^ Genetic alterations have been recently discovered in *IDH* genes, encoding isocitrate dehydrogenases 1 (IDH1) and 2 (IDH2).^13^ *IDH1* alterations are present in secondary GBM, but are rarely found in primary GBM; this difference allowed to discriminate between these two tumors types.^14, 15^ Mutations in *IDH1/IDH2* are of real diagnostic value because they also allow the confirmation of a glial tumor, and the distinction between grade II gliomas and pilocytic astrocytomas (grade I).^15^ Indeed, these mutations have not been detected in any pilocytic astrocytoma, suggesting that these tumors proceed from another mechanism. Over 70% of low-grade gliomas present mutations in *IDH1* or *IDH2*.^15^ On the contrary, only 5% of primary GBM are affected and 80% of secondary GBM have a mutated *IDH1*.^16^ The last WHO classification version incorporates new entities to divide GBM into IDH-wild-type GBM (90% of cases that corresponds to primary GBM) and IDH-mutant GBM (10% of cases that corresponds to secondary GBM).^2^

As for histological characteristics, GBM consists of two distinct tumor areas:

- A very high cell density area associated with atypical nuclei and a high mitotic index. The tissue consists only of tumor cells with the presence of newly formed blood vessels (micro-angiogenesis).
- An area in which isolated cancer cells invade the parenchyma that remains morphologically and functionally intact and devoid of newly formed blood vessels.^17^ The isolated cancer cells reflect the infiltrating nature of GBM; they are characterized by atypical and elongated nuclei.^3^

Necrotic areas are found in GBM, which correspond to hypoxic regions surrounded by highly anaplastic cells.^3^

## Hypoxia in GBM

Hypoxia means a low O_2_ partial pressure (1<pO_2_<10 mm Hg). It therefore constitutes a limitation on the availability of O_2_ in many diseases,^18^ such as brain and cardiovascular ischemia, diabetes and atherosclerosis. Hypoxia is also detected in solid cancers, particularly, in brain tumors such as GBM. In this type of tumors, hypoxic areas can be explained in different ways. First, the uncontrolled proliferation of cancer cells results in the formation of vessel-remote and highly hypoxic areas. Cancer cells react to this low partial pressure of O_2_ by stimulating neovascularization (from pre-existing blood vessels). Indeed, hypoxic cells secrete VEGF (vascular endothelial growth factor). After binding to its receptor on the surface of endothelial cells, VEGF promotes the building of new vessels that help to provide the tumor cells with the required amount of O_2_ and nutrients, thus ensuring their survival.^19^ This also leads to their dissemination in the body via the newly formed blood vessels rendered permeable by the VEGF signaling.^20^ Nevertheless, this tumor vascular network can in turn contribute to the formation of hypoxic areas. In fact, neovascularization gives rise to abnormal, too small, occluded vessels, or capillaries or arteriovenous bifurcations (imperfect vascularization).^21^ The result is an insufficient supply of O_2_, which maintains the hypoxic areas, and constitute a selection pressure that favors the rise of highly aggressive cells. Indeed, hypoxia-resistant cells trigger highly conserved signaling pathways to overcome the stress. Not only can these cells adapt to low O_2_,^22^ but they also resist anticancer treatments.^23^

**Figure 1:**
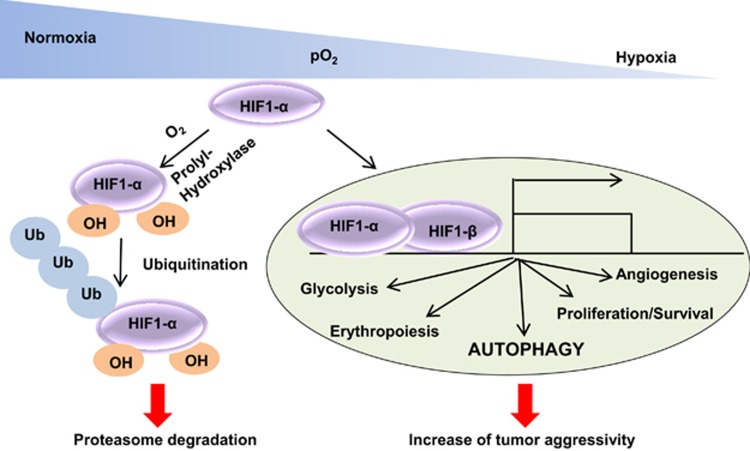
Hypoxia stabilizes HIF1 and promotes cell survival. In normoxia, HIF1-*α* is hydroxylated and then degraded by the proteasome. However in hypoxia, the absence of dioxygen leads to HIF1-*α* stabilization and complexation with HIF1-*β*. The HIF1*α*/HIF1-*β* complex is transcriptionally active, and following the binding of various coactivators, can target specific genes including those involved in autophagy, survival, proliferation and angiogenesis

The response and the adaptation of cells to hypoxia are controlled by transcription factors activated by a low partial pressure of O_2_. The most important transcription factor in this homeostasis is the hypoxia-inducible factor 1 or HIF1 (Figure 1). It consists of a heterodimeric protein composed of one *α* and one *β* subunit, which present some sequence and structural domain homologies. These two subunits are differentially expressed.^24, 25^ HIF1*β* is constitutively expressed regardless of the availability of O_2_.^26^ In contrast, HIF1*α* has a short half-life (*t*_½_~5 min) and is highly regulated by O_2_.^27^ In normoxia, HIF1*α* is subjected to hydroxylation, which allows recruitment of the von Hippel–Lindau protein, polyubiquitination and further degradation by the proteasome.^28^ However, in hypoxia, HIF1*α* is stabilized and transferred from the cytosol to the nucleus, where it dimerizes with HIF1*β*. The HIF1*α*/HIF1-*β* assembly becomes transcriptionally active,^26^ and several coactivators may join this complex to target and refine the transcription of specific genes, such as p300/CBP (CREB (cAMP response element-binding)-binding protein), TIF2 (transcription intermediary factor 2) and SRC-1 (steroid receptor coactivator-1).^29^ It has also been reported that HIF1-*α* can be influenced by mutations in mitochondrial enzymes. Among these, IDH1 that is essentially mutated in secondary GBM,^16^ has been correlated with an increase in HIF1-*α* protein level and transcriptional activity in GBM cell lines.^30^ However, this positive link has to be considered in relation with the tumor grade. Indeed, a recent study showed that the expression of HIF1A, as well as its downstream targets, is significantly reduced in grade II (low-grade diffuse) and III (anaplastic) gliomas harboring mutated IDH1/IDH2.^31^

At present, >100 genes have been identified as targets of HIF1.^32^ When expressed, these genes allow the cells to cope with hypoxia,^33^ they promote angiogenesis (VEGF)^34^ and cell survival (insulin-like growth factor-2),^35^ they boost glucose metabolism (glucose transporter-1,3—GLUT1,3)^36^ and facilitate tumor invasion (matrix metalloproteinases—MMPs).^37^

Hypoxia may also induce an intracellular recycling mechanism, called autophagy, allowing cancer cells to resist the hypoxic stress.^38, 39^

## Autophagy

Autophagy is a catabolic mechanism highly conserved during evolution. It is found in yeast, plants, worms, flies, mice and humans.^40^ Autophagy is currently a focus of major scientific interest.^41, 42^ Besides the proteasome, which mainly degrades short-lived proteins, autophagy eliminates, via lysosomes, altered organelles, long-lived and misfolded proteins. Autophagy occurs in response to nutrient starvation or oxidative stress, and leads to the formation of metabolic precursors (amino acids and fatty acids) and ATP, hence ensuring homeostasis and cell survival.^43^ However, inadequate repairs or major stress can lead to cell death, named autophagic death.^44^

**Figure 2:**
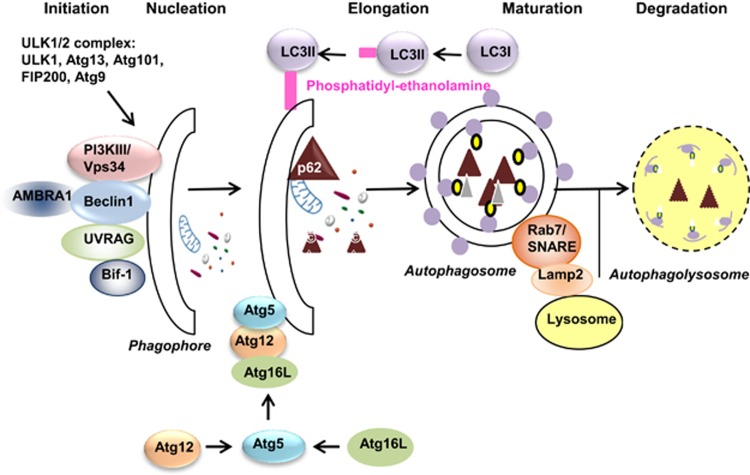
Autophagy process and molecular actors. Several external stimuli activate the ULK1/2 complex, which initiates isolation membrane near material to be removed. After this initiation step, the nucleation relies on the association of Beclin1 with class III phosphatidyl-inositol 3 kinase (PI3K III) to initiate phagophore formation. Other proteins like UVRAG, Bif-1 and AMBRA1 can also join this complex. The Atg5-Atg12-Atg16L complex and LC3 are two conjugation systems promoting the elongation of the phagophore to form the autophagosome, a vesicle containing the autophagic cargos. Cytosolic-free LC3, named LC3I, is transformed into LC3II, which consists of LC3 covalently bound to a phosphatidyl-ethanolamine molecule. LC3II is then anchored to the pre-autophagosomal membrane. After the binding of certain cytosolic cargos, the p62 protein leads them to the autophagosome, facilitating their degradation by autophagy. Thereafter, the autophagosome fuses with a lysosome to form the autophagolysosome, then the autophagic cargos are degraded by the lysosomal enzymes. Until its degradation, the p62 protein remains associated with the autophagosome

Different types of autophagy are currently described: microautophagy, macroautophagy and chaperone-mediated autophagy.^45^ Macroautophagy is the major recycling process of cytosolic components through the lysosomal pathway and is referred to as ‘autophagy' in the review (Figure 2). It appears from isolated membranes (or phagophore) that rearrange dynamically. This results in a double-membrane structure called autophagosome,^46^ which may include a portion of cytoplasm. The autophagosomal membrane probably originates from an infolding of the endoplasmic reticulum (ER) membrane.^47^ At the end of the process, the autophagosome fuses with a lysosome to form the autophagolysosome where the sequestered material is degraded by the lysosomal acid hydrolases.^48, 49^ The process provides metabolic precursors after their release into the cytosol by membrane permeases. The molecular machinery is orchestrated by the autophagy-related genes (*ATG*) family and autophagy-related proteins (Atg).^50^ These genes and proteins have been first characterized in the yeast *Saccharomyces cerevisiae*.^51^ In mammals, many genes (>30) are involved in the genetic control of autophagy. Proteins are classified into three subgroups: the ATG1/ULK1 (Unc-51-like autophagy-activating kinase 1) complex and its regulators, the Beclin1/phosphatidyl-inositol-3 kinase class III complex, and the Atg5-Atg12 and LC3 (light chain 3) conjugation systems.

- The ATG1/ULK1 complex is also called the initiator complex of autophagy. It is regulated by the mTOR protein (mammalian target of rapamycin), a kinase forming two complexes, named mTORC (mTOR complex)1 and mTORC2. In nutrient-rich conditions, mTORC1 phosphorylates and inhibits the ATG1/ULK1 complex, thus preventing the initiation of autophagy.^52^ In nutritional deficiency conditions or after the addition of rapamycin (mTORC1 inhibitor), mTORC1 dissociates from the complex, decreasing the phosphorylation level of the ATG1/ULK1 complex that becomes active^53^ and initiates autophagy.^54^ The mTORC2 complex (rapamycin insensitive) can also inhibit autophagy via activation of Akt. The latter activates mTORC1, but also phosphorylates and inhibits FoxO3 (forkhead box O3; reviewed in Hay^55^) that normally activates the transcription of important autophagy genes, such as *BNIP3* (B-cell lymphoma 2 (Bcl-2)/adenovirus E1B 19 kDa interacting protein 3) and *LC3* genes.^56^
- Formation of the Beclin1/class III phosphatidyl-inositide 3 kinase (PI3K) complex ensures autophagosome nucleation.^47^ Beclin1 activation requires its release from Bcl-2 and/or Bcl-x_L_ (Bcl-2 homolog X long protein).^57^ Indeed, Beclin1 is inactive when it is bound to these proteins.^58^ Other proteins such as UVRAG (UV radiation resistance-associated gene protein),^59^ Bif-1^60^ and AMBRA1 (activating molecule in Beclin1-regulated autophagy)^61^ could interact with Beclin1 to participate in autophagy processing and/or regulation.
- The ubiquitin-like Atg5-Atg12 and LC3 conjugation systems are necessary for autophagosome elongation.^62^ After its conjugation in the cytosol, the Atg5-Atg12-Atg16L complex transiently binds to the pre-autophagosomal membrane^62^ and facilitates the recruitment of the MAP-LC3 (microtubule-associated protein 1 (MAP1) LC3) protein or LC3.^63^ The free form of the latter (LC3I) is transformed into the lipidated form LC3II by the covalent binding of a molecule of phosphatidyl ethanolamine.^64, 65^

This modification is needed to anchor LC3 to the pre-autophagosomal membrane.^64, 66^ LC3II remains bound to the autophagosome until its degradation (Figure 2).^67^ This conversion of LC3I to LC3II is a key marker of autophagy, more particularly when the autophagic flux is inhibited. Indeed, a functional autophagy must lead to the degradation of cargos, which can be evaluated in the presence of lysosomal inhibitors such as chloroquine (CQ) or bafilomycin A1. These inhibitors induce autophagosome and LC3II accumulation in the case of an efficient and functional autophagy. However, evidencing autophagy induction requires that autophagic flux is inhibited, otherwise LC3II would be rapidly degraded and recycled.^41^

The p53 protein is another important actor of the regulation of autophagy; according to its localization, it can activate or inhibit autophagy. The cytosolic p53 inhibits the formation of autophagic vacuoles^68^ by interaction with FIP200 (FAK (focal adhesion kinase) family kinase-interacting protein of 200 kDa), whereas nuclear p53 acts as a transcriptional factor of genes implied in autophagy regulation. For example, PTEN, which is an important negative regulator of the Akt/mTOR pathway.^69^ The p53-dependent expression of the lysosomal protein DRAM (damage-regulated autophagy modulator) activates macroautophagy.^70, 71, 72^ Moreover, we have already shown that the efficiency of the signaling is dependent on its genetic profile (wild type or mutated). Indeed, p53 does not always induce autophagy as we evidenced in neuroblastoma cell lines in response to cobalt chloride (CoCl_2_, a hypoxia-mimicking agent). In the wild-type *TP53* neuroblastoma cell line (SHSY5Y), CoCl_2_ induced an apoptotic signaling involving p53, characterized by a decrease in mitochondrial membrane potential, and activation of cysteinyl aspartate-specific proteinases (caspases) 9 and 3. In the *TP53*-mutated neuroblastoma cell line or in the wild-type cells with invalidated *TP53* expression (by small hairpin RNA (shRNA)), CoCl_2_ induced an autophagic signaling. It precedes cell death, occurring later in the absence of caspase activation, but with the release of the mitochondrial apoptosis-inducing factor, favoring an autophagic cell death.^73^ Another regulator example is AMPK (adenosine monophosphate (AMP)-activated protein kinase), whose role will be detailed below in the section ‘Autophagy and Hypoxia', as this protein is being activated under hypoxia condition.^74^

## Autophagy and GBM

Today, the data on the inhibition and/or the development of a tumor via autophagy seem contradictory. Autophagy presents a dual function in cancer.^75, 76^ In the early stages of tumorigenesis, it would limit cell proliferation, DNA damage and therefore tumor progression. When the tumor size increases, its blood supply is restricted, and autophagy may promote the survival of tumor cells in the nutrient-deficient and hypoxic tumor regions.^77^ Furthermore, autophagy, as already evoked, may lead to either cell survival or death.

In particular, it has been shown that *BECN1*, the gene encoding Beclin1, is deleted in 75%, 50% and 40% in ovarian, breast and prostate cancers, respectively.^78^ The mutation of a single allele of *BECN1* increases the incidence of tumors.^79^ Deletion of *ATG7* has also been reported in several cancers.^80, 81^ In metabolic stress conditions, autophagy inhibition realized by inactivation of *BECN1* or *ATG5* increases DNA damage and chromosomal instabilities.^82^ Thus, autophagy seems to prevent damage to DNA and limit the occurrence of cancers. Finally, the tumor suppressor protein, PTEN, induces an autophagic signaling, whereas it is inhibited by oncogenes.^83^ However, the autophagy ability to recycle the intracellular contents protects tumor cells from adverse environmental conditions. Thus, this signaling appears as an adaptive mechanism in poorly vascularized solid tumors. It ensures independence in nutrients and energy necessary for the survival of tumor cells in hypoxic regions.^84^ Indeed, as already described above, hypoxia causes the stabilization of HIF1*α*. This activates the transcription of several target genes such as *VEGF*, leading to tumor neovascularization^34^ and *GLUT1-3*, ensuring the transport of glucose.^36^

These contradictory aspects are also observed in the case of GBM. Indeed, autophagy can positively or negatively regulate both GBM invasion and resistance to therapy, the two hallmarks of GBM aggressiveness.^85^ Concerning invasion and cell motility, whereas Catalano *et al.*^86^ reported an impairment of migration and invasion of GBM cells after autophagy induction, Palumbo *et al.*^87^ noticed this impairment after autophagy inhibition, especially when EGFR was coinhibited, thus highlighting the importance of growth factor signaling.

When considering therapy resistance, the interplay between classical chemotherapy and autophagy regulation seems to be complex. For example, it has been recently shown that curcurbitacin I, a potent anticancer molecule, induced an autophagy process in GBM cells and in a xenograft model, leading to cell survival and tumor protection.^88^ However, itraconazole was shown to inhibit the development of GBM through an autophagic signaling induction, whereas the blockage of autophagy significantly reverses the antiproliferative effects of this molecule, suggesting an antitumor role of autophagy in response to itraconazole.^89^ Temozolomide (TMZ) (Temodal, MERCK, Kirkland, Quebec, Canada) is the most used drug to treat GBM; it induces autophagy in GBM cell lines and provides them with resistance to the treatment. As early as 2004, Kanzawa *et al.*^90^ have reported an induction of survival autophagy in GBM cell lines, suppressing the antitumor effect of TMZ. The use of 3-MA (3-methyladenine, class III PI3K inhibitor) or bafilomycin A1 restored TMZ cytotoxicity, suggesting that autophagy inhibition, combined to TMZ in GBM treatment, could enhance the cytotoxicity of this chemotherapeutic agent.^91^ This twin treatment is currently the most promising strategy to limit tumor development.^92, 93, 94, 95, 96, 97^ Indeed, CQ and its analog hydroxychloroquine (HCQ), which are the only autophagy flux inhibitors^98^ approved by the US Food and Drug Administration (FDA),^99^ are in a phase III clinical trial in adjuvant settings, following surgery, chemotherapy and radiotherapy. CQ-treated patients benefit from a clearly better median survival, compared with placebo-treated patients.^100^ Despite the promising effect of CQ,^100^ a recent phase I/II clinical trial combining HCQ with TMZ and radiation therapy, in patients with newly diagnosed GBM showed that the maximum tolerated dose of HCQ was unable to consistently inhibit autophagy and that dose-limiting toxicity prevented the possibility of moving to higher doses of HCQ.^101^ Such a study highlights the toxic side effect of this molecule, used in combination with classical chemotherapeutic agents.^102^ As a result, we are waiting for further development of novel autophagy inhibitors with less cytotoxicity.

In a contradictory way, another approach to GBM treatment could consist in targeting the autophagy inhibitor mTOR.^42, 103^ Indeed, dysregulation of the PI3K/Akt/mTOR signaling pathway is common in human cancers, inducing uncontrolled cell proliferation, which can be targeted by rapamycin analogs. However, even if in GBM, the presence of autophagy inducers such as RAD001 (mTOR inhibitor) allowed TMZ to induce an autophagic death signaling,^104^ only few cancers responded to such treatment. It appears that mTOR inhibitors should be associated with other growth factor pathway inhibitors^103, 105^ or with autophagy inhibitors, considering they can also induce a survival autophagy. Thus, such twin treatment could counteract cell survival pathways, and at the same time, sensitize tumor cells to death. Furthermore, it has been shown that inhibition of mTOR could boost temozolomide-induced senescence, whereas autophagy inhibition triggered apoptosis and decreased senescence.^106^ Nevertheless, the link between autophagy and senescence needs to be clarified. Indeed, in a particular genetically engineered GBM mouse model in which autophagy was disrupted, gliomagenesis or GBM formation was significantly reduced or completely inhibited.^107^ *In vitro* studies showed an intact cellular growth, but a real senescence induction essentially characterized by activation of retinoblastoma 1 (RB1/p105) and an increase in CDKN1B/p27 level.^107, 108, 109, 110^ Thus, the induction of senescence, consecutively to autophagy dysregulation, needs to be investigated more thoroughly to constitute a hopeful therapy able to reduce GBM aggressiveness.

Finally, targeting autophagic process could also interfere with antiangiogenic therapy, frequently used in cancer. For example, the treatment of GBM cell lines with CQ in combination with 3-MA, ATG7 and BECN1 small interfering RNA (siRNA), blocked the autophagy induced by ZD6474, a small molecule that blocks the VEGF receptor (VEGFR) activity, thereby sensitizing GBM cells to ZD6474-induced apoptosis.^111^ Indeed, GBM are characterized by their high capacity of angiogenesis^112^ that is important for tumor growth, making antiangiogenic molecules crucial for GBM treatment.^113^ However, owing to their tumor devascularization capacity, these molecules cause increased intratumoral hypoxia, which in turn activates autophagy and finally help tumor cells to resist treatment.^114^ Thus, the antitumor effects of antiangiogenic agents seem to be enhanced when they are combined with autophagy inhibitors.^115^

## Autophagy and Hypoxia

**Figure 3:**
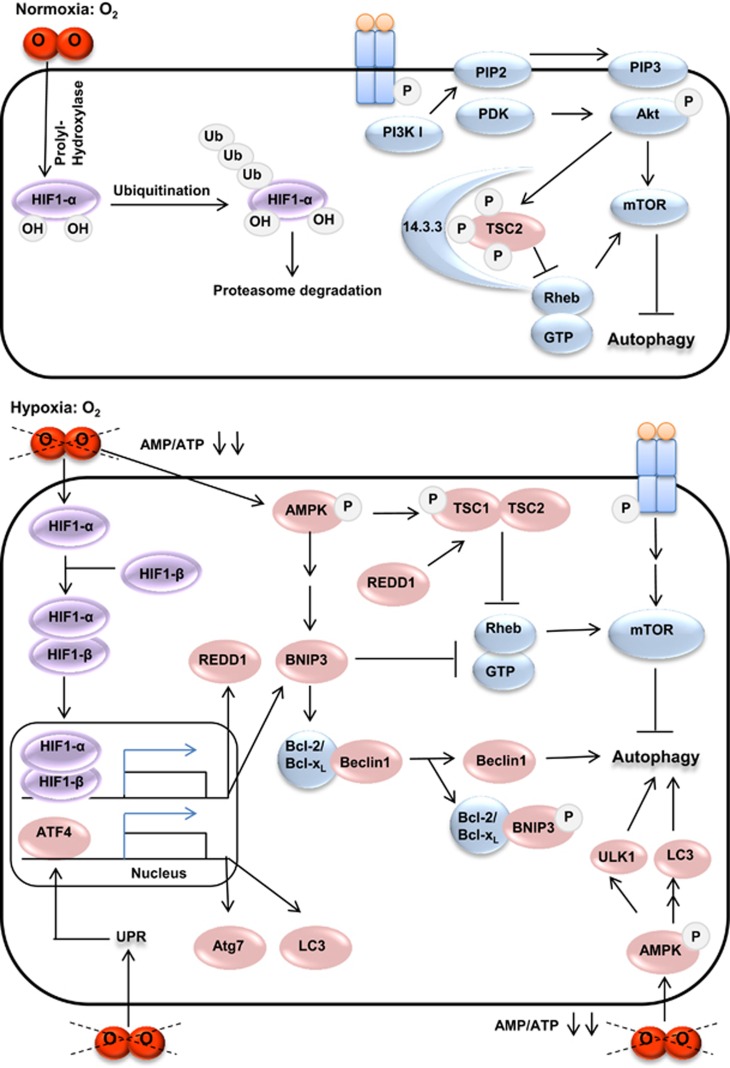
Different actors involved in the induction of autophagy by hypoxia. In normoxia, HIF1-*α* is hydroxylated and degraded by proteasome. As the presence of dioxygen is favorable to cellular proliferation, receptor tyrosine kinases that promote proliferation are activated, leading to mTOR activation and autophagy inhibition. However, in hypoxia, HIF1-*α* is stabilized and it associates with HIF1-*β* to form HIF1 that activates the transcription of several genes whose encoded proteins activate autophagy. For example, REDD1 activates the mTOR inhibitors, thus removing the autophagy inhibition. BNIP3 can also activate autophagy by decreasing the RHEB/GTP level. Moreover, BNIP3 is a HIF1 target, and it competes with Beclin1 for binding to Bcl-2 or Bcl-x_L_, triggering the Beclin1 release and the induction of autophagy. BNIP3 can also be induced by AMPK that is activated in response to hypoxia. AMPK can also activate ULK1 and LC3, thus triggering autophagy. In hypoxic conditions, the unfolded protein response (UPR) activates the transcription factor, ATF4, which in turn activates Atg7 and LC3, two proteins involved in the autophagosome elongation

Several studies have reported a link between hypoxia and the induction of autophagy as summarized in Figure 3. In fact, the hypoxia factor HIF1 activates the transcription of the *BNIP3* gene,^116^ encoding the BNIP3 protein, which can induce autophagy. Indeed, BNIP3 displaces Beclin1 from Bcl-2 or Bcl-x_L_ by competing for the same binding site; free Beclin1 then induces autophagy.^57^ BNIP3 can also activate autophagy by inhibiting the RHEB (Ras homolog enriched in brain) protein, whose activity is required for mTORC1 activation.^117^

In addition, the energy depletion caused by hypoxia increases the intracellular AMP/ATP ratio, which stimulates the monophosphate-activated protein kinase or AMPK, an energy-sensing switch.^118^ AMPK indirectly inhibits mTOR by phosphorylation and activation of TSC2 (tuberous sclerosis complex 2) and Raptor, inhibiting the activity of mTORC1 and activating autophagy.^119^ Indeed, the TSC1–TSC2 complex contains a GTP (guanosine triphosphate)ase-activating protein domain, which stimulates the GTPase activity of RHEB and thereby preventing it to activate mTORC1.^120^ In addition, AMPK regulates autophagy by phosphorylating ULK1^121, 122, 123^ and thus inducing mTOR-independent autophagy. AMPK also promotes the accumulation of FoxO3 in the nucleus and the expression of its autophagy-associated target genes (*LC3* and *BNIP3*).^124^ Moreover, AMPK has been shown to be an important regulator of the HIF1 activity and of the expression of its target genes in prostate cancer cells cultured in the presence of CoCl_2_.^125^ Not only is the TSC1–TSC2 complex maintained in its active form (phosphorylation of TSC1) by AMPK, but it is also activated by the REDD1 (regulated in development and DNA damage responses 1) protein, itself activated under hypoxic condition; then autophagy is induced by preventing TSC2 phosphorylation and sequestration by the 14-3-3 protein.^126^ An interesting study on GBM cell lines showed that hypoxia induced autophagy.^115^ This signaling was preceded by a stabilization of HIF1*α* and an increase in phospho-AMPK (active AMPK). The extinction of one of these proteins by siRNA significantly reduced the autophagic process. Hypoxia-induced autophagy was also dependent on BNIP3, whose expression increases under hypoxic condition in GBM cell lines and in xenograft-derived cells. Inhibition of autophagy with CQ induced caspase-dependent apoptotic death, without affecting BNIP3 expression. Moreover, this hypoxia-induced autophagy promoted the tumor resistance to antiangiogenic therapy. Indeed, when GBM xenografts were treated with bevacizumab alone, an increase in BNIP3 expression and hypoxia-associated growth were recorded. Addition of the autophagy inhibitor CQ blocked this process. *In vivo, ATG7* extinction in athymic mice by shRNA, also disrupted tumor growth especially when combined with bevacizumab treatment.^115^ Another hypoxia-activated protein, the tumor suppressor protein PML (promyelocytic leukemia) activates autophagy by binding mTOR, thereby preventing its interaction with RHEB, causing its deactivation and its nuclear accumulation.^127, 128^

Furthermore, under hypoxic conditions, the protein trafficking (secretion or incorporation in the plasma membrane) is defected because O_2_ is required for protein maturation.^129^ Hypoxia initiates the unfolded protein response or UPR, which can in turn induce autophagy through ATF4 (activating transcription factor 4) activation.^130^ Indeed, ATF4, stabilized in hypoxia, is able to upregulate the LC3B protein, which is essential for the autophagic process. The inhibition of ATF4-activated autophagy significantly increases apoptosis and reduces cell viability in several hypoxic cancer cells.^130^ Another study carried out on other cancer cells, including U373 MG, a GBM cell line, the hypoxia-induced UPR activated PERK (protein kinase RNA-like ER kinase) in the ER. The latter activates ATF4 and another transcription factor, the C/EBP (CCAAT/enhancer-binding protein) homologous protein or CHOP, which increases the expression of the *LC3B* and *ATG5* genes, thus inducing autophagy. Exposure to CQ sensitized cancer cells to hypoxia and xenografted tumors to irradiation.^131^

## Conclusion

The impact of autophagy on GBM cell fate remains ambiguous. Autophagy could protect cancer cells from drastic environmental conditions such as hypoxia, and from chemo and radiotherapy, but can also inhibit the cell growth and induce senescence, depending on the context. Autophagy induced by chemotherapeutic agents may either promote or inhibit cell survival, depending on the drug used. As for the hypoxia-induced autophagy in this tumor, it has been shown that autophagy was able to protect cancer cells against hypoxia, but also could enhance their resistance to antiangiogenic therapy.^115^ Furthermore, recent clinical studies have been conducted with CQ, which inhibits autophagy, or with mTOR inhibitors, which activates this process; both kinds of therapies resulted in beneficial effects on GBM evolution.^42, 102^ These facts highlight the importance of autophagy modulation as a potent anti-GBM strategy that could improve the efficiency of chemotherapy.^115^ Thus, more studies are needed to elucidate the exact role of autophagy in GBM and to delineate the associated therapy.

## Figures and Tables

**Table 1:** Percentage of mutations of particular genes in primary and secondary glioblastomas

**Mutated genes**	**Primary glioblastoma**	**Secondary glioblastoma**	**References**
*EGFR*	36%	8%	11
*p16INK4a*	31%	19%	11
*PTEN*	25%	4%	11
*TP53*	30%	50%	11
*IDH1*	5%	80%	14, 15, 16

The difference in the occurrence of certain mutations between the two types of glioblastomas allowed its molecular classification. Whereas primary glioblastoma is mainly characterized by mutations in *EGFR*, *p16INK4a* and *PTEN*, secondary glioblastoma harbors *TP53* and *IDH1* mutations.

## Abbreviations

AMP: adenosine monophosphate
AMPK: AMP-activated protein kinase
ATF4: activating transcription factor 4
*ATG*: autophagy-related genes
Atg: autophagy-related proteins
ATP: adenosine triphosphate
Bcl-2: B-cell lymphoma 2
Bcl-x_L_: Bcl-2 homolog X long protein
***BECN1***: gene encoding Beclin1
BNIP3: Bcl-2/adenovirus E1B 19 kDa interacting protein 3
Caspase: cysteinyl aspartate-specific proteinase
CHOP: C/EBP homologous protein
CoCl_2_: cobalt chloride
CQ: chloroquine
EGFR: epidermal growth factor receptor
ER: endoplasmic reticulum
FoxO3: forkhead box O3
GBM: glioblastoma multiforme
GLUT1,3: glucose transporter-1,3
GTP: guanosine triphosphate
HCQ: hydroxychloroquine
HIF1: hypoxia-inducible factor 1
IDH: isocitrate dehydrogenase
LC3: light chain 3
3-MA: 3-methyladenine
mTOR: mammalian target of rapamycin
mTORC: mTOR complex
O_2_: dioxygen
p16InK4a: p16 cyclin-dependent kinase inhibitor 4a
PI3K: phosphatidyl-inositide 3 kinase
PML: promyelocytic leukemia
PTEN: phosphatase and tensin
REDD1: regulated in development and DNA damage responses 1
RHEB: Ras homolog enriched in brain
shRNA: small hairpin RNA
siRNA: small interfering RNA
*TP53*: gene encoding p53
TSC1/TSC2: tuberous sclerosis complex 1 and 2
ULK1: Unc-51-like autophagy-activating kinase 1
UPR: unfolded protein response
UVRAG: UV radiation resistance-associated gene protein
VEGF: vascular endothelial growth factor
WHO: World Health Organization

## References

[bib1] Louis DN, Ohgaki H, Wiestler OD, Cavenee WK, Burger PC, Jouvet A et al. The 2007 WHO classification of tumours of the central nervous system. Acta Neuropathol 2007; 114: 97–109. 17618441 10.1007/s00401-007-0243-4 PMC1929165

[bib2] Louis DN, Perry A, Reifenberger G, von Deimling A, Figarella-Branger D, Cavenee WK et al. The 2016 World Health Organization Classification of Tumors of the Central Nervous System: a summary. Acta Neuropathol 2016; 131: 803–820. 27157931 10.1007/s00401-016-1545-1

[bib3] Figarella-Branger D, Colin C, Coulibaly B, Quilichini B, Maues De Paula A, Fernandez C et al. [Histological and molecular classification of gliomas]. Rev Neurol 2008; 164: 505–515. 18565348 10.1016/j.neurol.2008.03.011

[bib4] Furnari FB, Fenton T, Bachoo RM, Mukasa A, Stommel JM, Stegh A et al. Malignant astrocytic glioma: genetics, biology, and paths to treatment. Genes Dev 2007; 21: 2683–2710. 17974913 10.1101/gad.1596707

[bib5] Wen PY, Kesari S. Malignant gliomas in adults. N Engl J Med 2008; 359: 492–507. 18669428 10.1056/NEJMra0708126

[bib6] Polivka J, Polivka J, Rohan V, Pesta M, Repik T, Pitule P et al. Isocitrate dehydrogenase-1 mutations as prognostic biomarker in glioblastoma multiforme patients in West Bohemia. Biomed Res Int 2014; 2014: 735659. 24511544 10.1155/2014/735659 PMC3910481

[bib7] Sturm D, Bender S, Jones DTW, Lichter P, Grill J, Becher O et al. Paediatric and adult glioblastoma: multiform (epi)genomic culprits emerge. Nat Rev Cancer 2014; 14: 92–107. 24457416 10.1038/nrc3655 PMC4003223

[bib8] Glioblastome, origine, causes, diagnostic, pronostic, traitements, vaccins. http://gfme.free.fr/maladie/glioblastome.html.

[bib9] Krex D, Klink B, Hartmann C, von Deimling A, Pietsch T, Simon M et al. Long-term survival with glioblastoma multiforme. Brain J Neurol 2007; 130: 2596–2606.. 10.1093/brain/awm204 17785346

[bib10] Ohgaki H, Kleihues P. Genetic pathways to primary and secondary glioblastoma. Am J Pathol 2007; 170: 1445–1453. 17456751 10.2353/ajpath.2007.070011 PMC1854940

[bib11] Ohgaki H, Dessen P, Jourde B, Horstmann S, Nishikawa T, Di Patre PL et al. Genetic pathways to glioblastoma: a population-based study. Cancer Res 2004; 64: 6892–6899. 15466178 10.1158/0008-5472.CAN-04-1337

[bib12] Fujisawa H, Reis RM, Nakamura M, Colella S, Yonekawa Y, Kleihues P et al. Loss of heterozygosity on chromosome 10 is more extensive in primary (*de novo* than in secondary glioblastomas. Lab Invest 2000; 80: 65–72. 10653004 10.1038/labinvest.3780009

[bib13] Sanson M, Marie Y, Paris S, Idbaih A, Laffaire J, Ducray F et al. Isocitrate dehydrogenase 1 codon 132 mutation is an important prognostic biomarker in gliomas. J Clin Oncol 2009; 27: 4150–4154. 19636000 10.1200/JCO.2009.21.9832

[bib14] Parsons DW, Jones S, Zhang X, Lin JC, Leary RJ, Angenendt P et al. An integrated genomic analysis of human glioblastoma multiforme. Science 2008; 321: 1807–1812. 18772396 10.1126/science.1164382 PMC2820389

[bib15] Yan H, Bigner DD, Velculescu V, Parsons DW. Mutant metabolic enzymes are at the origin of gliomas. Cancer Res 2009; 69: 9157–9159v. 19996293 10.1158/0008-5472.CAN-09-2650 PMC2794981

[bib16] Nobusawa S, Watanabe T, Kleihues P, Ohgaki H. IDH1 mutations as molecular signature and predictive factor of secondary glioblastomas. Clin Cancer Res 2009; 15: 6002–6007. 19755387 10.1158/1078-0432.CCR-09-0715

[bib17] Figarella-Branger D, Bouvier C. [Histological classification of human gliomas: state of art and controversies]. Bull Cancer 2005; 92: 301–309. 15888386

[bib18] Höckel M, Vaupel P. Biological consequences of tumor hypoxia. Semin Oncol 2001; 28: 36–41. 11395851

[bib19] Ferrara N. VEGF and the quest for tumour angiogenesis factors. Nat Rev Cancer 2002; 2: 795–803. 12360282 10.1038/nrc909

[bib20] Box C, Rogers SJ, Mendiola M, Eccles SA. Tumour-microenvironmental interactions: paths to progression and targets for treatment. Semin Cancer Biol 2010; 20: 128–138. 20599506 10.1016/j.semcancer.2010.06.004

[bib21] Shchors K, Evan G. Tumor angiogenesis: cause or consequence of cancer? Cancer Res 2007; 67: 7059–7061. 17671171 10.1158/0008-5472.CAN-07-2053

[bib22] Papandreou I, Powell A, Lim AL, Denko N. Cellular reaction to hypoxia: sensing and responding to an adverse environment. Mutat Res 2005; 569: 87–100. 15603754 10.1016/j.mrfmmm.2004.06.054

[bib23] Kaur B, Khwaja FW, Severson EA, Matheny SL, Brat DJ, Van Meir EG. Hypoxia and the hypoxia-inducible-factor pathway in glioma growth and angiogenesis. Neuro Oncol 2005; 7: 134–153. 15831232 10.1215/S1152851704001115 PMC1871894

[bib24] Déry M-AC, Michaud MD, Richard DE. Hypoxia-inducible factor 1: regulation by hypoxic and non-hypoxic activators. Int J Biochem Cell Biol 2005; 37: 535–540. 15618010 10.1016/j.biocel.2004.08.012

[bib25] Zagórska A, Dulak J. HIF-1: the knowns and unknowns of hypoxia sensing. Acta Biochim Pol 2004; 51: 563–585. 15448722

[bib26] Kallio PJ, Pongratz I, Gradin K, McGuire J, Poellinger L. Activation of hypoxia-inducible factor 1alpha: posttranscriptional regulation and conformational change by recruitment of the Arnt transcription factor. Proc Natl Acad Sci US 1997; 94: 5667–5672. 10.1073/pnas.94.11.5667 PMC20836 9159130

[bib27] Salceda S, Caro J. Hypoxia-inducible factor 1alpha (HIF-1alpha) protein is rapidly degraded by the ubiquitin-proteasome system under normoxic conditions. Its stabilization by hypoxia depends on redox-induced changes. J Biol Chem 1997; 272: 22642–22647. 9278421 10.1074/jbc.272.36.22642

[bib28] Wang GL, Jiang BH, Semenza GL. Effect of altered redox states on expression and DNA-binding activity of hypoxia-inducible factor 1. Biochem Biophys Res Commun 1995; 212: 550–556. 7626069 10.1006/bbrc.1995.2005

[bib29] Carrero P, Okamoto K, Coumailleau P, O'Brien S, Tanaka H, Poellinger L. Redox-regulated recruitment of the transcriptional coactivators CREB-binding protein and SRC-1 to hypoxia-inducible factor 1alpha. Mol Cell Biol 2000; 20: 402–415. 10594042 10.1128/mcb.20.1.402-415.2000 PMC85095

[bib30] Zhao S, Lin Y, Xu W, Jiang W, Zha Z, Wang P et al. Glioma-derived mutations in IDH1 dominantly inhibit IDH1 catalytic activity and induce HIF-1alpha. Science 2009; 324: 261–265. 19359588 10.1126/science.1170944 PMC3251015

[bib31] Kickingereder P, Sahm F, Radbruch A, Wick W, Heiland S, Deimling Av et al. IDH mutation status is associated with a distinct hypoxia/angiogenesis transcriptome signature which is non-invasively predictable with rCBV imaging in human glioma. Sci Rep 2015; 5: 16238. 26538165 10.1038/srep16238 PMC4633672

[bib32] Ke Q, Costa M. Hypoxia-inducible factor-1 (HIF-1). Mol Pharmacol 2006; 70: 1469–1480. 16887934 10.1124/mol.106.027029

[bib33] Semenza GL. HIF-1 mediates metabolic responses to intratumoral hypoxia and oncogenic mutations. J Clin Invest 2013; 123: 3664–3671. 23999440 10.1172/JCI67230 PMC3754249

[bib34] Levy AP, Levy NS, Wegner S, Goldberg MA. Transcriptional regulation of the rat vascular endothelial growth factor gene by hypoxia. J Biol Chem 1995; 270: 13333–13340. 7768934 10.1074/jbc.270.22.13333

[bib35] Feldser D, Agani F, Iyer NV, Pak B, Ferreira G, Semenza GL. Reciprocal positive regulation of hypoxia-inducible factor 1alpha and insulin-like growth factor 2. Cancer Res 1999; 59: 3915–3918. 10463582

[bib36] Chen C, Pore N, Behrooz A, Ismail-Beigi F, Maity A. Regulation of glut1 mRNA by hypoxia-inducible factor-1. Interaction between H-ras and hypoxia. J Biol Chem 2001; 276: 9519–9525. 11120745 10.1074/jbc.M010144200

[bib37] Ben-Yosef Y, Lahat N, Shapiro S, Bitterman H, Miller A. Regulation of endothelial matrix metalloproteinase-2 by hypoxia/reoxygenation. Circ Res 2002; 90: 784–791. 11964371 10.1161/01.res.0000015588.70132.dc

[bib38] Frieboes HB, Huang JS, Yin WC, McNally LR. Chloroquine-mediated cell death in metastatic pancreatic adenocarcinoma through inhibition of autophagy. JOP 2014; 15: 189–197. 24618445 10.6092/1590-8577/1900

[bib39] Wilkinson S, O'Prey J, Fricker M, Ryan KM. Hypoxia-selective macroautophagy and cell survival signaled by autocrine PDGFR activity. Genes Dev 2009; 23: 1283–1288. 19487569 10.1101/gad.521709 PMC2701580

[bib40] Levine B, Yuan J. Autophagy in cell death: an innocent convict? J Clin Invest 2005; 115: 2679–2688. 16200202 10.1172/JCI26390 PMC1236698

[bib41] Klionsky DJ, Abdelmohsen K, Abe A, Abedin MJ, Abeliovich H, Acevedo Arozena A et al. Guidelines for the use and interpretation of assays for monitoring autophagy (3rd edition). Autophagy 2016; 12: 1–222. 26799652 10.1080/15548627.2015.1100356 PMC4835977

[bib42] Rebecca VW, Amaravadi RK. Emerging strategies to effectively target autophagy in cancer. Oncogene 2016; 35: 1–11. 25893285 10.1038/onc.2015.99 PMC4838040

[bib43] Codogno P, Meijer AJ. Autophagy and signaling: their role in cell survival and cell death. Cell Death Differ 2005; 12(Suppl 2): 1509–1518. 16247498 10.1038/sj.cdd.4401751

[bib44] Azad MB, Chen Y, Henson ES, Cizeau J, McMillan-Ward E, Israels SJ et al. Hypoxia induces autophagic cell death in apoptosis-competent cells through a mechanism involving BNIP3. Autophagy 2008; 4: 195–204. 18059169 10.4161/auto.5278 PMC3164855

[bib45] Cuervo AM, Stefanis L, Fredenburg R, Lansbury PT, Sulzer D. Impaired degradation of mutant alpha-synuclein by chaperone-mediated autophagy. Science 2004; 305: 1292–1295. 15333840 10.1126/science.1101738

[bib46] Juhasz G, Neufeld TP. Autophagy: a forty-year search for a missing membrane source. PLoS Biol 2006; 4: e36. 16464128 10.1371/journal.pbio.0040036 PMC1363699

[bib47] Axe EL, Walker SA, Manifava M, Chandra P, Roderick HL, Habermann A et al. Autophagosome formation from membrane compartments enriched in phosphatidylinositol 3-phosphate and dynamically connected to the endoplasmic reticulum. J Cell Biol 2008; 182: 685–701. 18725538 10.1083/jcb.200803137 PMC2518708

[bib48] Levine B, Klionsky DJ. Development by self-digestion: molecular mechanisms and biological functions of autophagy. Dev Cell 2004; 6: 463–477. 15068787 10.1016/s1534-5807(04)00099-1

[bib49] Homma K, Suzuki K, Sugawara H. The Autophagy Database: an all-inclusive information resource on autophagy that provides nourishment for research. Nucleic Acids Res 2011; 39: D986–D990. 20972215 10.1093/nar/gkq995 PMC3013813

[bib50] Klionsky DJ, Cregg JM, Dunn WA, Emr SD, Sakai Y, Sandoval IV et al. A unified nomenclature for yeast autophagy-related genes. Dev Cell 2003; 5: 539–545. 14536056 10.1016/s1534-5807(03)00296-x

[bib51] Mizushima N. Autophagy: process and function. Genes Dev 2007; 21: 2861–2873. 18006683 10.1101/gad.1599207

[bib52] Ravikumar B, Sarkar S, Davies JE, Futter M, Garcia-Arencibia M, Green-Thompson ZW et al. Regulation of mammalian autophagy in physiology and pathophysiology. Physiol Rev 2010; 90: 1383–1435. 20959619 10.1152/physrev.00030.2009

[bib53] Hosokawa N, Hara T, Kaizuka T, Kishi C, Takamura A, Miura Y et al. Nutrient-dependent mTORC1 association with the ULK1-Atg13-FIP200 complex required for autophagy. Mol Biol Cell 2009; 20: 1981–1991. 19211835 10.1091/mbc.E08-12-1248 PMC2663915

[bib54] Yeh Y-Y, Wrasman K, Herman PK. Autophosphorylation within the Atg1 activation loop is required for both kinase activity and the induction of autophagy in *Saccharomyces cerevisiae*. Genetics 2010; 185: 871–882. 20439775 10.1534/genetics.110.116566 PMC2907206

[bib55] Hay N. Interplay between FOXO, TOR, and Akt. Biochim Biophys Acta 2011; 1813: 1965–1970. 21440577 10.1016/j.bbamcr.2011.03.013 PMC3427795

[bib56] Singh A, Ye M, Bucur O, Zhu S, Tanya Santos M, Rabinovitz I et al. Protein phosphatase 2A reactivates FOXO3a through a dynamic interplay with 14-3-3 and AKT. Mol Biol Cell 2010; 21: 1140–1152. 20110348 10.1091/mbc.E09-09-0795 PMC2836964

[bib57] Sridharan S, Jain K, Basu A. Regulation of autophagy by kinases. Cancers 2011; 3: 2630–2654. 24212825 10.3390/cancers3022630 PMC3757434

[bib58] Pattingre S, Tassa A, Qu X, Garuti R, Liang XH, Mizushima N et al. Bcl-2 antiapoptotic proteins inhibit Beclin 1-dependent autophagy. Cell 2005; 122: 927–939. 16179260 10.1016/j.cell.2005.07.002

[bib59] Liang C, Feng P, Ku B, Oh B-H, Jung JU. UVRAG: a new player in autophagy and tumor cell growth. Autophagy 2007; 3: 69–71. 17106237 10.4161/auto.3437

[bib60] Takahashi Y, Coppola D, Matsushita N, Cualing HD, Sun M, Sato Y et al. Bif-1 interacts with Beclin 1 through UVRAG and regulates autophagy and tumorigenesis. Nat Cell Biol 2007; 9: 1142–1151. 17891140 10.1038/ncb1634 PMC2254521

[bib61] Fimia GM, Stoykova A, Romagnoli A, Giunta L, Di Bartolomeo S, Nardacci R et al. Ambra1 regulates autophagy and development of the nervous system. Nature 2007; 447: 1121–1125. 17589504 10.1038/nature05925

[bib62] Mizushima N, Kuma A, Kobayashi Y, Yamamoto A, Matsubae M, Takao T et al. Mouse Apg16L, a novel WD-repeat protein, targets to the autophagic isolation membrane with the Apg12-Apg5 conjugate. J Cell Sci 2003; 116: 1679–1688. 12665549 10.1242/jcs.00381

[bib63] Hanada T, Noda NN, Satomi Y, Ichimura Y, Fujioka Y, Takao T et al. The Atg12-Atg5 conjugate has a novel E3-like activity for protein lipidation in autophagy. J Biol Chem 2007; 282: 37298–37302. 17986448 10.1074/jbc.C700195200

[bib64] Tanida I, Ueno T, Kominami E. LC3 and autophagy. Methods Mol Biol 2008; 445: 77–88. 18425443 10.1007/978-1-59745-157-4_4

[bib65] Lee Y-K, Lee J-A. Role of the mammalian ATG8/LC3 family in autophagy: differential and compensatory roles in the spatiotemporal regulation of autophagy. BMB Rep 2016; 49: 424–430. 27418283 10.5483/BMBRep.2016.49.8.081 PMC5070729

[bib66] Kirisako T, Ichimura Y, Okada H, Kabeya Y, Mizushima N, Yoshimori T et al. The reversible modification regulates the membrane-binding state of Apg8/Aut7 essential for autophagy and the cytoplasm to vacuole targeting pathway. J Cell Biol 2000; 151: 263–276. 11038174 10.1083/jcb.151.2.263 PMC2192639

[bib67] Kabeya Y, Mizushima N, Ueno T, Yamamoto A, Kirisako T, Noda T et al. LC3, a mammalian homologue of yeast Apg8p, is localized in autophagosome membranes after processing. EMBO J 2000; 19: 5720–5728. 11060023 10.1093/emboj/19.21.5720 PMC305793

[bib68] Tasdemir E, Chiara Maiuri M, Morselli E, Criollo A, D'Amelio M, Djavaheri-Mergny M et al. A dual role of p53 in the control of autophagy. Autophagy 2008; 4: 810–814. 18604159 10.4161/auto.6486

[bib69] Feng Z, Hu W, de Stanchina E, Teresky AK, Jin S, Lowe S et al. The regulation of AMPK beta1, TSC2, and PTEN expression by p53: stress, cell and tissue specificity, and the role of these gene products in modulating the IGF-1-AKT-mTOR pathways. Cancer Res 2007; 67: 3043–3053. 17409411 10.1158/0008-5472.CAN-06-4149

[bib70] Criollo A, Dessen P, Kroemer G. DRAM: a phylogenetically ancient regulator of autophagy. Cell Cycle 2009; 8: 2319–2320. 19633415 10.4161/cc.8.15.9153

[bib71] Mah LY, O'Prey J, Baudot AD, Hoekstra A, Ryan KM. DRAM-1 encodes multiple isoforms that regulate autophagy. Autophagy 2012; 8: 18–28. 22082963 10.4161/auto.8.1.18077 PMC3335989

[bib72] Yoon J-H, Her S, Kim M, Jang I-S, Park J. The expression of damage-regulated autophagy modulator 2 (DRAM2) contributes to autophagy induction. Mol Biol Rep 2012; 39: 1087–1093. 21584698 10.1007/s11033-011-0835-x

[bib73] Naves T, Jawhari S, Jauberteau M-O, Ratinaud M-H, Verdier M. Autophagy takes place in mutated p53 neuroblastoma cells in response to hypoxia mimetic CoCl(2). Biochem Pharmacol 2013; 85: 1153–1161. 23380477 10.1016/j.bcp.2013.01.022

[bib74] Liu L, Cash TP, Jones RG, Keith B, Thompson CB, Simon MC. Hypoxia-induced energy stress regulates mRNA translation and cell growth. Mol Cell 2006; 21: 521–531. 16483933 10.1016/j.molcel.2006.01.010 PMC3153113

[bib75] White E, DiPaola RS. The double-edged sword of autophagy modulation in cancer. Clin Cancer Res 2009; 15: 5308–5316. 19706824 10.1158/1078-0432.CCR-07-5023 PMC2737083

[bib76] Zhong Z, Sanchez-Lopez E, Karin M. Autophagy, inflammation, and immunity: A Troika Governing Cancer and Its Treatment. Cell 2016; 166: 288–298. 27419869 10.1016/j.cell.2016.05.051 PMC4947210

[bib77] Liu EY, Ryan KM. Autophagy and cancer–issues we need to digest. J Cell Sci 2012; 125: 2349–2358. 22641689 10.1242/jcs.093708

[bib78] Aita VM, Liang XH, Murty VV, Pincus DL, Yu W, Cayanis E et al. Cloning and genomic organization of beclin 1, a candidate tumor suppressor gene on chromosome 17q21. Genomics 1999; 59: 59–65. 10395800 10.1006/geno.1999.5851

[bib79] Yue Z, Jin S, Yang C, Levine AJ, Heintz N. Beclin 1, an autophagy gene essential for early embryonic development, is a haploinsufficient tumor suppressor. Proc Natl Acad Sci USA 2003; 100: 15077–15082. 14657337 10.1073/pnas.2436255100 PMC299911

[bib80] Amaravadi R, Debnath J. Mouse models address key concerns regarding autophagy inhibition in cancer therapy. Cancer Discov 2014; 12: 873–875. 10.1158/2159-8290.CD-14-0618 PMC4124512 25092744

[bib81] Takamura A, Komatsu M, Hara T, Sakamoto A, Kishi C, Waguri S et al. Autophagy-deficient mice develop multiple liver tumors. Genes Dev 2011; 25: 795–800. 21498569 10.1101/gad.2016211 PMC3078705

[bib82] Tsuchihara K, Fujii S, Esumi H. Autophagy and cancer: dynamism of the metabolism of tumor cells and tissues. Cancer Lett 2009; 278: 130–138. 19004545 10.1016/j.canlet.2008.09.040

[bib83] Maiuri MC, Tasdemir E, Criollo A, Morselli E, Vicencio JM, Carnuccio R et al. Control of autophagy by oncogenes and tumor suppressor genes. Cell Death Differ 2009; 16: 87–93. 18806760 10.1038/cdd.2008.131

[bib84] Jin S, White E. Role of autophagy in cancer: management of metabolic stress. Autophagy 2007; 3: 28–31. 16969128 10.4161/auto.3269 PMC2770734

[bib85] Galavotti S, Bartesaghi S, Faccenda D, Shaked-Rabi M, Sanzone S, McEvoy A et al. The autophagy-associated factors DRAM1 and p62 regulate cell migration and invasion in glioblastoma stem cells. Oncogene 2013; 32: 699–712. 22525272 10.1038/onc.2012.111

[bib86] Catalano M, D'Alessandro G, Lepore F, Corazzari M, Caldarola S, Valacca C et al. Autophagy induction impairs migration and invasion by reversing EMT in glioblastoma cells. Mol Oncol 2015; 9: 1612–1625. 26022108 10.1016/j.molonc.2015.04.016 PMC5528793

[bib87] Palumbo S, Tini P, Toscano M, Allavena G, Angeletti F, Manai F et al. Combined EGFR and autophagy modulation impairs cell migration and enhances radiosensitivity in human glioblastoma cells. J Cell Physiol 2014; 229: 1863–1873. 24691646 10.1002/jcp.24640

[bib88] Yuan G, Yan S-F, Xue H, Zhang P, Sun J-T, Li G. Cucurbitacin I induces protective autophagy in glioblastoma *in vitro* and *in vivo*. J Biol Chem 2014; 289: 10607–10619. 24599950 10.1074/jbc.M113.528760 PMC4036180

[bib89] Liu R, Li J, Zhang T, Zou L, Chen Y, Wang K et al. Itraconazole suppresses the growth of glioblastoma through induction of autophagy: involvement of abnormal cholesterol trafficking. Autophagy 2014; 10: 1241–1255. 24905460 10.4161/auto.28912 PMC4203550

[bib90] Kanzawa T, Germano IM, Komata T, Ito H, Kondo Y, Kondo S. Role of autophagy in temozolomide-induced cytotoxicity for malignant glioma cells. Cell Death Differ 2004; 11: 448–457. 14713959 10.1038/sj.cdd.4401359

[bib91] Zou Y, Wang Q, Li B, Xie B, Wang W. Temozolomide induces autophagy via ATM-AMPK-ULK1 pathways in glioma. Mol Med Rep 2014; 10: 411–416. 24737504 10.3892/mmr.2014.2151

[bib92] Manic G, Obrist F, Kroemer G, Vitale I, Galluzzi L. Chloroquine and hydroxychloroquine for cancer therapy. Mol Cell Oncol 2014; 1: e29911. 27308318 10.4161/mco.29911 PMC4905171

[bib93] Choi DS, Blanco E, Kim Y-S, Rodriguez AA, Zhao H, Huang TH-M et al. Chloroquine eliminates cancer stem cells through deregulation of Jak2 and DNMT1. Stem Cells 2014; 32: 2309–2323. 24809620 10.1002/stem.1746 PMC4138251

[bib94] Balic A, Sørensen MD, Trabulo SM, Sainz B, Cioffi M, Vieira CR et al. Chloroquine targets pancreatic cancer stem cells via inhibition of CXCR4 and hedgehog signaling. Mol Cancer Ther 2014; 13: 1758–1771. 24785258 10.1158/1535-7163.MCT-13-0948

[bib95] Zhao X, Sun R, Yang X, Liu D, Lei D, Jin T et al. Chloroquine-enhanced efficacy of cisplatin in the treatment of hypopharyngeal carcinoma in xenograft mice. PLoS One 2015; 10: e0126147. 25923669 10.1371/journal.pone.0126147 PMC4414471

[bib96] Rangwala R, Leone R, Chang YC, Fecher LA, Schuchter LM, Kramer A et al. Phase I trial of hydroxychloroquine with dose-intense temozolomide in patients with advanced solid tumors and melanoma. Autophagy 2014; 10: 1369–1379. 24991839 10.4161/auto.29118 PMC4203514

[bib97] Sui X, Kong N, Wang X, Fang Y, Hu X, Xu Y et al. JNK confers 5-fluorouracil resistance in p53-deficient and mutant p53-expressing colon cancer cells by inducing survival autophagy. Sci Rep 2014; 4: 4694. 24733045 10.1038/srep04694 PMC3986705

[bib98] Gonzalez-Noriega A, Grubb JH, Talkad V, Sly WS. Chloroquine inhibits lysosomal enzyme pinocytosis and enhances lysosomal enzyme secretion by impairing receptor recycling. J Cell Biol 1980; 85: 839–852. 7190150 10.1083/jcb.85.3.839 PMC2111452

[bib99] Hu Y-L, Jahangiri A, Delay M, Aghi MK. Tumor cell autophagy as an adaptive response mediating resistance to treatments such as antiangiogenic therapy. Cancer Res 2012; 72: 4294–4299. 22915758 10.1158/0008-5472.CAN-12-1076 PMC3432684

[bib100] Sotelo J, Briceño E, López-González MA. Adding chloroquine to conventional treatment for glioblastoma multiforme: a randomized, double-blind, placebo-controlled trial. Ann Intern Med 2006; 144: 337–343. 16520474 10.7326/0003-4819-144-5-200603070-00008

[bib101] Rosenfeld MR, Ye X, Supko JG, Desideri S, Grossman SA, Brem S et al. A phase I/II trial of hydroxychloroquine in conjunction with radiation therapy and concurrent and adjuvant temozolomide in patients with newly diagnosed glioblastoma multiforme. Autophagy 2014; 10: 1359–1368. 24991840 10.4161/auto.28984 PMC4203513

[bib102] Wojton J, Meisen WH, Kaur B. How to train glioma cells to die: molecular challenges in cell death. J Neurooncol 2016; 126: 377–384. 26542029 10.1007/s11060-015-1980-1 PMC5283073

[bib103] Dufour M, Dormond-Meuwly A, Demartines N, Dormond O. Targeting the mammalian target of rapamycin (mTOR) in cancer therapy: lessons from past and future perspectives. Cancers 2011; 3: 2478–2500. 24212820 10.3390/cancers3022478 PMC3757428

[bib104] Josset E, Burckel H, Noël G, Bischoff P. The mTOR inhibitor RAD001 potentiates autophagic cell death induced by temozolomide in a glioblastoma cell line. Anticancer Res 2013; 33: 1845–1851. 23645729

[bib105] Nghiemphu PL, Lai A, Green RM, Reardon DA, Cloughesy T. A dose escalation trial for the combination of erlotinib and sirolimus for recurrent malignant gliomas. J Neurooncol 2012; 110: 245–250. 22918789 10.1007/s11060-012-0960-y PMC3472078

[bib106] Filippi-Chiela EC, Bueno e Silva MM, Thomé MP, Lenz G. Single-cell analysis challenges the connection between autophagy and senescence induced by DNA damage. Autophagy 2015; 11: 1099–1113. 25701485 10.1080/15548627.2015.1009795 PMC4590630

[bib107] Gammoh N, Fraser J, Puente C, Syred HM, Kang H, Ozawa T et al. Suppression of autophagy impedes glioblastoma development and induces senescence. Autophagy 2016; 12: 1431–1439. 27304681 10.1080/15548627.2016.1190053 PMC5082770

[bib108] Acosta JC, Banito A, Wuestefeld T, Georgilis A, Janich P, Morton JP et al. A complex secretory program orchestrated by the inflammasome controls paracrine senescence. Nat Cell Biol 2013; 15: 978–990. 23770676 10.1038/ncb2784 PMC3732483

[bib109] Alexander K, Hinds PW. Requirement for p27(KIP1) in retinoblastoma protein-mediated senescence. Mol Cell Biol 2001; 21: 3616–3631. 11340156 10.1128/MCB.21.11.3616-3631.2001 PMC86983

[bib110] Muñoz-Espín D, Serrano M. Cellular senescence: from physiology to pathology. Nat Rev Mol Cell Biol 2014; 15: 482–496. 24954210 10.1038/nrm3823

[bib111] Shen J, Zheng H, Ruan J, Fang W, Li A, Tian G et al. Autophagy inhibition induces enhanced proapoptotic effects of ZD6474 in glioblastoma. Br J Cancer 2013; 109: 164–171. 23799852 10.1038/bjc.2013.306 PMC3708568

[bib112] Gagner J-P, Law M, Fischer I, Newcomb EW, Zagzag D. Angiogenesis in gliomas: imaging and experimental therapeutics. Brain Pathol 2005; 15: 342–363. 16389946 10.1111/j.1750-3639.2005.tb00119.x PMC8095871

[bib113] Chamberlain MC. Bevacizumab for the treatment of recurrent glioblastoma. Clin Med Insights Oncol 2011; 5: 117–129. 21603247 10.4137/CMO.S7232 PMC3095028

[bib114] Hu Y-L, Jahangiri A, De Lay M, Aghi MK. Hypoxia-induced tumor cell autophagy mediates resistance to anti-angiogenic therapy. Autophagy 2012; 8: 979–981. 22714142 10.4161/auto.20232 PMC3427265

[bib115] Hu Y-L, DeLay M, Jahangiri A, Molinaro AM, Rose SD, Carbonell WS et al. Hypoxia-induced autophagy promotes tumor cell survival and adaptation to antiangiogenic treatment in glioblastoma. Cancer Res 2012; 72: 1773–1783. 22447568 10.1158/0008-5472.CAN-11-3831 PMC3319869

[bib116] Azad MB, Gibson SB. Role of BNIP3 in proliferation and hypoxia-induced autophagy: implications for personalized cancer therapies. Ann N Y Acad Sci 2010; 1210: 8–16. 20973794 10.1111/j.1749-6632.2010.05778.x

[bib117] Li Y, Wang Y, Kim E, Beemiller P, Wang C-Y, Swanson J et al. Bnip3 mediates the hypoxia-induced inhibition on mammalian target of rapamycin by interacting with Rheb. J Biol Chem 2007; 282: 35803–35813. 17928295 10.1074/jbc.M705231200

[bib118] Hardie DG. Sensing of energy and nutrients by AMP-activated protein kinase. Am J Clin Nutr 2011; 93: 891S–896S. 21325438 10.3945/ajcn.110.001925

[bib119] Gwinn DM, Shackelford DB, Egan DF, Mihaylova MM, Mery A, Vasquez DS et al. AMPK phosphorylation of raptor mediates a metabolic checkpoint. Mol Cell 2008; 30: 214–226. 18439900 10.1016/j.molcel.2008.03.003 PMC2674027

[bib120] Inoki K, Zhu T, Guan K-L. TSC2 mediates cellular energy response to control cell growth and survival. Cell 2003; 115: 577–590. 14651849 10.1016/s0092-8674(03)00929-2

[bib121] Egan DF, Shackelford DB, Mihaylova MM, Gelino S, Kohnz RA, Mair W et al. Phosphorylation of ULK1 (hATG1) by AMP-activated protein kinase connects energy sensing to mitophagy. Science 2011; 331: 456–461. 21205641 10.1126/science.1196371 PMC3030664

[bib122] Kim J, Kundu M, Viollet B, Guan K-L. AMPK and mTOR regulate autophagy through direct phosphorylation of Ulk1. Nat Cell Biol 2011; 13: 132–141. 21258367 10.1038/ncb2152 PMC3987946

[bib123] Lee JW, Park S, Takahashi Y, Wang H-G. The association of AMPK with ULK1 regulates autophagy. PLoS One 2010; 5: e15394. 21072212 10.1371/journal.pone.0015394 PMC2972217

[bib124] Chiacchiera F, Simone C. Inhibition of p38alpha unveils an AMPK-FoxO3A axis linking autophagy to cancer-specific metabolism. Autophagy 2009; 5: 1030–1033. 19587525 10.4161/auto.5.7.9252

[bib125] Lee M, Hwang J-T, Lee H-J, Jung S-N, Kang I, Chi S-G et al. AMP-activated protein kinase activity is critical for hypoxia-inducible factor-1 transcriptional activity and its target gene expression under hypoxic conditions in DU145 cells. J Biol Chem 2003; 278: 39653–39661. 12900407 10.1074/jbc.M306104200

[bib126] Brugarolas J, Lei K, Hurley RL, Manning BD, Reiling JH, Hafen E et al. Regulation of mTOR function in response to hypoxia by REDD1 and the TSC1/TSC2 tumor suppressor complex. Genes Dev 2004; 18: 2893–2904. 15545625 10.1101/gad.1256804 PMC534650

[bib127] Bernardi R, Guernah I, Jin D, Grisendi S, Alimonti A, Teruya-Feldstein J et al. PML inhibits HIF-1alpha translation and neoangiogenesis through repression of mTOR. Nature 2006; 442: 779–785. 16915281 10.1038/nature05029

[bib128] Bernardi R, Papa A, Egia A, Coltella N, Teruya-Feldstein J, Signoretti S et al. Pml represses tumour progression through inhibition of mTOR. EMBO Mol Med 2011; 3: 249–257. 21387562 10.1002/emmm.201100130 PMC3123467

[bib129] Rzymski T, Milani M, Singleton DC, Harris AL. Role of ATF4 in regulation of autophagy and resistance to drugs and hypoxia. Cell Cycle 2009; 8: 3838–3847. 19887912 10.4161/cc.8.23.10086

[bib130] Rzymski T, Milani M, Pike L, Buffa F, Mellor HR, Winchester L et al. Regulation of autophagy by ATF4 in response to severe hypoxia. Oncogene 2010; 29: 4424–4435. 20514020 10.1038/onc.2010.191

[bib131] Rouschop KMA, van den Beucken T, Dubois L, Niessen H, Bussink J, Savelkouls K et al. The unfolded protein response protects human tumor cells during hypoxia through regulation of the autophagy genes MAP1LC3B and ATG5. J Clin Invest 2010; 120: 127–141. 20038797 10.1172/JCI40027 PMC2798689

